# Controlling the oxidation and wettability of liquid metal via femtosecond laser for high-resolution flexible electronics

**DOI:** 10.3389/fchem.2022.965891

**Published:** 2022-09-01

**Authors:** Jingzhou Zhang, Chengjun Zhang, Haoyu Li, Yang Cheng, Qing Yang, Xun Hou, Feng Chen

**Affiliations:** ^1^ State Key Laboratory for Manufacturing System Engineering and Shaanxi Key Laboratory of Photonics Technology for Information, School of Electronic Science and Engineering, Xi’an Jiaotong University, Xi’an, China; ^2^ School of Mechanical Engineering, Xi’an Jiaotong University, Xi’an, China

**Keywords:** oxide-EGaIn, femtosecond laser, wettability, flexible electronics, electronic skin

## Abstract

Liquid metal-based electronic devices are attracting increasing attention owing to their excellent flexibility and high conductivity. However, a simple way to realize liquid metal electronics on a microscale without photolithography is still challenging. Herein, the wettability and adhesion of liquid metal are controlled by combining the stirring method, femtosecond laser microfabrication, and sacrificial layer assistant. The adhesive force of liquid metal is dramatically enhanced by adjusting its oxidation. The wetting area is limited to a micro-pattern by a femtosecond laser and sacrificial layer. On this basis, a high-resolution liquid metal printing method is proposed. The printing resolution can be controlled even less than 50 μm. The resultant liquid metal pattern is applied to electronic skin, which shows uniformity, flexibility, and stability. It is anticipated that this liquid metal printing method will hold great promise in the fields of flexible electronics.

## 1 Introduction

Due to high electrical conductivity, excellent deformability, and ultralow toxicity, Ga-based liquid metal has received increasing attention (Daeneke et al., 2018; Kalantar-Zadeh et al., 2019; Chen et al., 2020; Malakooti et al., 2020; Sun et al., 2020; Zuraiqi et al., 2020; Liu et al., 2021) and has been widely applied to the field of flexible electronics, such as wearable devices (Zhang et al., 2020a; Liu et al., 2020; Yu et al., 2020), soft robots (Sheng et al., 2015; Zhang et al., 2015; Mao et al., 2020), electronic skin (Wang et al., 2019; Guo et al., 2020; Rao et al., 2020), and flexible sensors (Rahimi et al., 2018; Zhou et al., 2018; Wu et al., 2020; Wang et al., 2021; Zhou et al., 2021; Libanori et al., 2022; Zhao et al., 2022). Adhering liquid metal to a flexible substrate is the foundation of its application (Zhang et al., 2019; Zhu et al., 2019; Chang et al., 2020). Traditional microelectronics manufacturing technology mainly focuses on silicon-based substrates and lithography, which is not suitable for the fabrication of liquid metal-based electronics. Recently, various studies have explored printing liquid metal on a soft substrate, including direct writing (Guo et al., 2018; Peng et al., 2020), injection printing (Yang et al., 2017; Teng et al., 2019a; Teng et al., 2019b), mask printing (Kramer et al., 2013; Liu et al., 2019), photolithography (Hirsch et al., 2016; An et al., 2022), and magnetic patterning (Guo et al., 2019; Xu et al., 2019; Zhang et al., 2021). Nevertheless, when the printing resolution is less than 50 μm, the as-prepared liquid metal patterns usually lack uniformity and connectivity, which result from the significant liquidity and high surface tension of the liquid metal. Although photolithography is applied to realize high-resolution liquid metal patterns, this method usually requires a high cost and complicated technology.

Building wetting/dewetting surfaces by an ultrafast laser is utilized for forming and transferring liquid metal patterns (Joshipura et al., 2018; Pan et al., 2018; Zhang et al., 2020b; Yong et al., 2020), which not only owns remarkable flexibility but also presents high resolution. Controlling the surface morphology is the key to realizing a wetting/dewetting surface for liquid metal (Kramer et al., 2014; Babu et al., 2021; Johnston et al., 2022). For instance, Joshipura et al. (2018) proposed a rough spray-coating and laser scanning method to form liquid metal patterns. Silicon dioxide rough structures were pre-coated on printing substrates and then were selectively removed by a laser. Liquid metal could only adhere to the laser-scanned area, while other areas present low adhesion. Wu et al. (2020) used a laser-induced selective adhesion transfer method to fabricate liquid metal pressure sensors. Micro/nanostructures could be formed on a polymer surface by an ultrafast laser, showing low adhesion to liquid metal, while the untreated flat area presents high adhesion to liquid metal. However, current laser-induced wetting/dewetting substrates mainly focus on reducing the adhesion of liquid metal to the dewetting area rather than improving the adhesive force on the wetting area. So that these laser-induced printing methods are only suitable for some polymer substrates, and their printing resolution is hard to further improve. A simple way to improve the adhesion and resolution of liquid metal patterns by a laser scanning method is still challenging.

In this work, a liquid metal printing method is developed by combining oxide-EGaIn (O-EGaIn) and femtosecond (fs) laser microfabrication. The whole process does not need any dopants or printing masks. To enhance the wettability of liquid metal, EGaIn is partly oxidized and turned into O-EGaIn by simple magnetic stirring. The as-prepared liquid metal also presents better plasticity and maintains a high conductivity at the same time. To obtain the wetting/dewetting surface of liquid metal, an fs laser is utilized for selective surface ablation. Hierarchical nanostructures are decorated on laser-scanned areas, showing ultralow adhesion to O-EGaIn. The untreated area remains a high adhesion area for liquid metal printing. In addition, the sacrificial layer is proposed to further improve the printing resolution. On this basis, an O-EgaIn-based electronic patch with high uniformity has been successfully prepared.

## 2 Results and discussion

### 2.1 Fabrication of fs laser-induced liquid metal pattern with high resolution

**FIGURE 1:**
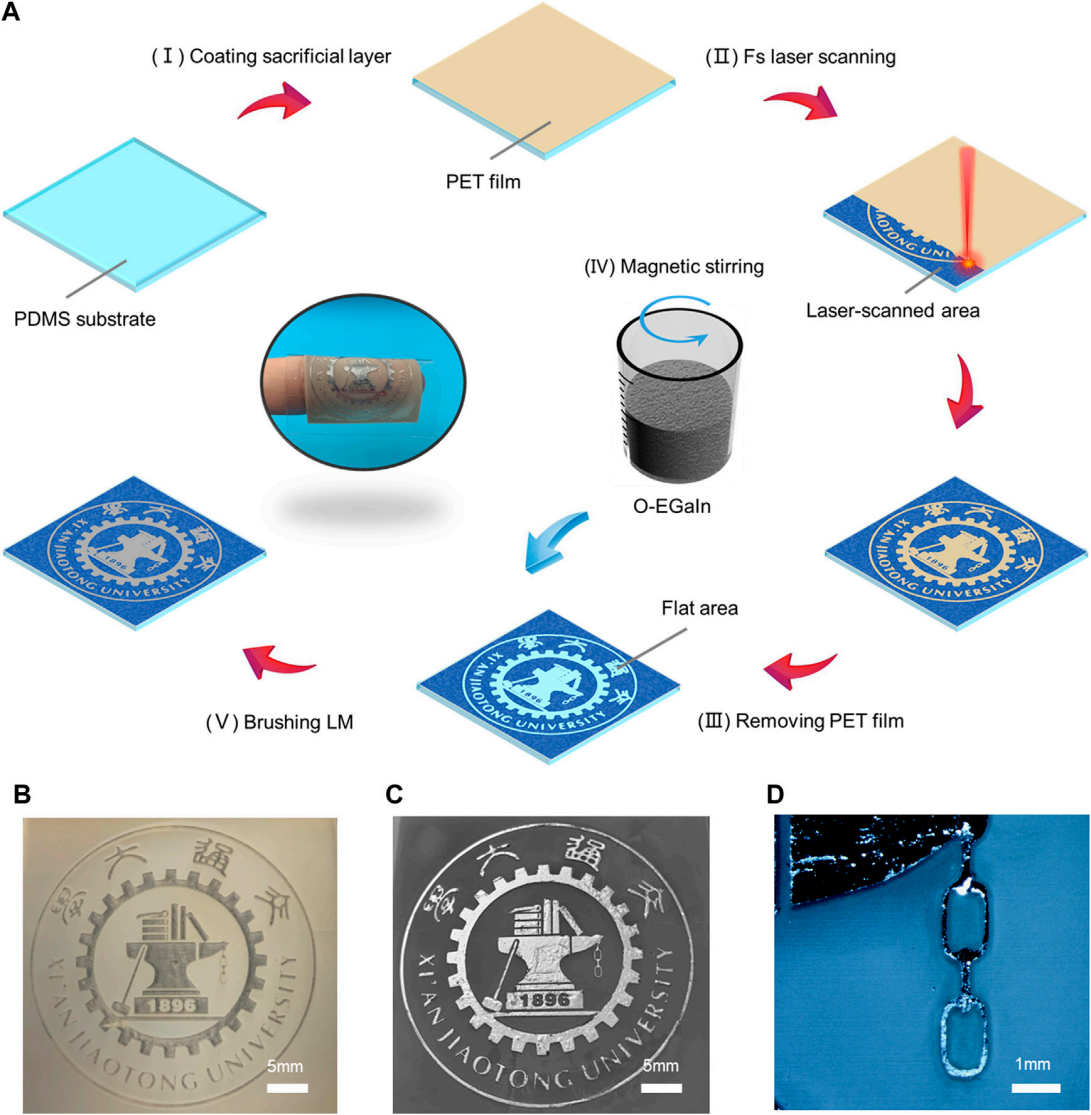
**(A)** Schematic diagram of high-resolution liquid metal printing method on PDMS surface. **(B)** Optical image of a logo of XJTU on PDMS substrate after fs laser selective ablation. **(C)** Photograph of the liquid metal logo on the resultant surface with high resolution. **(D)** Photograph of the liquid metal logo on the resultant surface with higher magnification.

Polydimethylsiloxane (PDMS) is chosen as the printing substrate on account of its flexibility, transparency, and biocompatibility (Zhang et al., 2020c). As illustrated in Figure 1A, the whole printing process can be divided into five steps. First, polyethylene terephthalate (PET) thin film (10 μm) is coated on the PDMS substrate, playing the role of the sacrificial layer (yellow area). Second, the resultant surface is selectively irradiated by an fs laser. To obtain the logo of Xian Jiaotong University (XJTU), the complementary area of the logo is ablated (Figure 1B). Because the thickness of the sacrificial layer is much less than the focused depth (Supplementary Figure S1), the sacrificial layer has no significant effect on the morphology of the laser-ablated area (Supplementary Figure S2). Micro/nanostructures will be built on the laser-irradiated area (dark blue area). Next, the surface is cleaned with alcohol in an ultrasonic bath for 10 min. The remaining PET pattern (yellow area) will completely fall off and a flat PDMS pattern (light blue area) will be exposed. Then, to facilitate liquid metal printing, liquid EGaIn is poured into a beaker and stirred by a magnetic stirrer for 60 min. In this way, O-EGaIn with higher adhesive force is obtained without any additional materials (Handschuh-Wang et al., 2020; Kong et al., 2020; Li et al., 2020) and chemical treatments. Finally, O-EGaIn is brushed on the as-prepared surface (gray area). Owing to the liquid-metal-repellence of the laser-ablated area, liquid metal can only adhere to the untreated area so that the liquid metal logo of XJTU is formed (Figure 1C). Figure 1D and Supplementary Figure S3 show the details of the liquid metal logo with higher magnification. Combining with the sacrificial layer assistant and O-EGaIn, the resolution of the laser printing pattern is reached up to 50 μm. Meanwhile, this laser printing method is also applicable to other polymer substrates and obtains various complex liquid metal patterns.

### 2.2 Property of O-EGaIn

**FIGURE 2:**
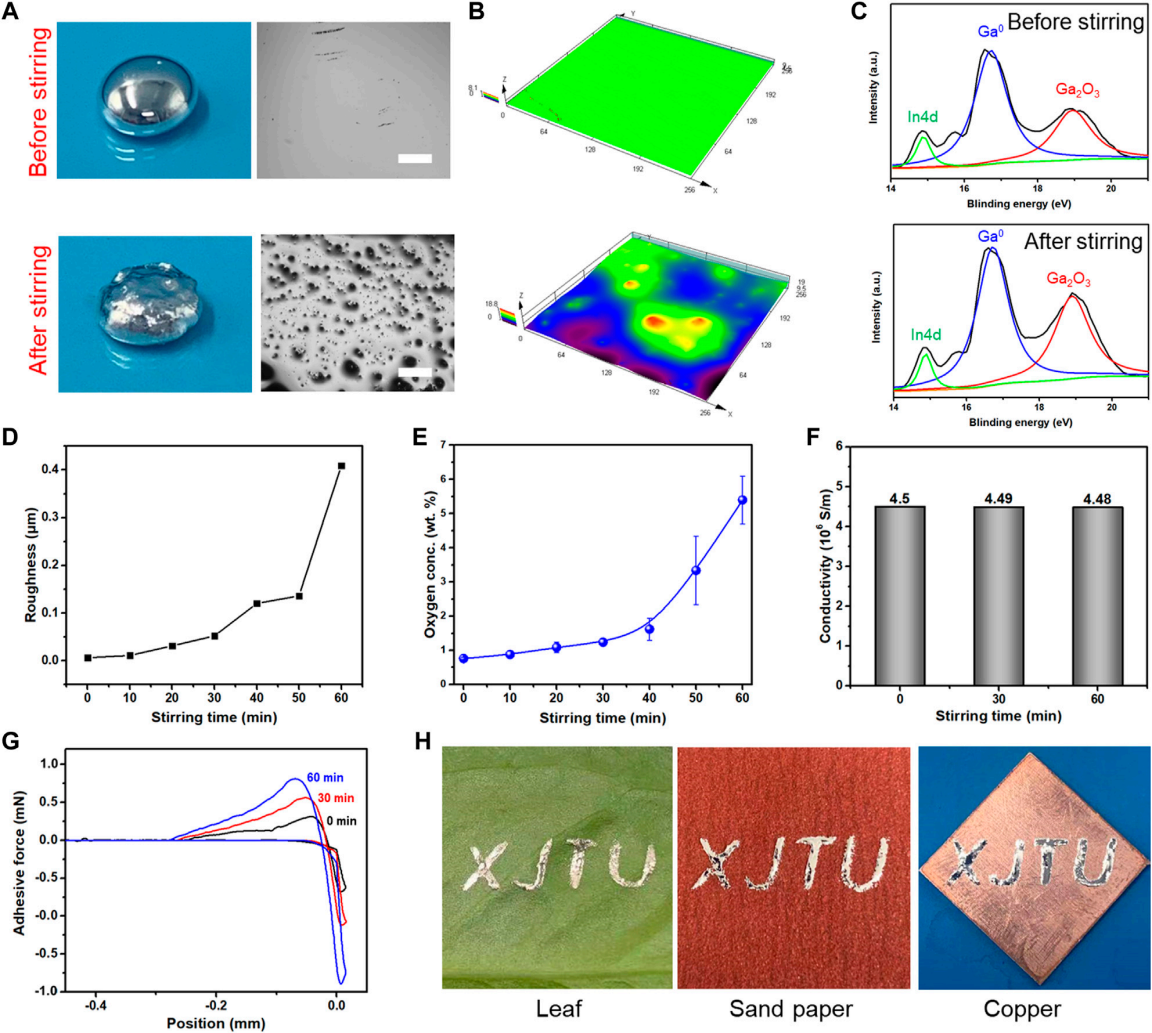
Comparison between original and magnetic stirred EGaIn droplets. **(A)** Photographs of the droplets and their surfaces, scale bar: 500 μm. **(B)** 3D confocal microscopy images of EGaIn and O-EGaIn surfaces. **(C)** XPS analysis of EGaIn and O-EGaIn surfaces. **(D)** Surface roughness of EGaIn with different magnetic stirring times. **(E)** Relationship between oxygen contents of EGaIn and magnetic stirring time. **(F)** Conductivity of EGaIn with different stirring times. **(G)** Adhesive force between flat PDMS surface and EGaIn droplet with different stirring times. **(H)** O-EGaIn as electronic direct ink writing on different surfaces.

In order to increase the adhesive force between liquid metal and PDMS substrate, EGaIn is stirred by a magnetic stirrer for 60 min. Figure 2A presents the liquid EGaIn droplets before and after stirring. The original EGaIn droplet has a typical core-shell structure, which includes liquid metal and an oxide shell. Owing to the high surface tension of liquid metal, the surface of the EGaIn droplet is atomically smooth, and the surface roughness is only 0.005 μm (Figure 2B). During magnetic stirring, the oxide layer of liquid metal is constantly broken, while a new oxide layer is constantly formed in an air environment. Cracked oxide layers gradually mix into liquid metal so that the surface of liquid metal becomes rough and its fluidity is getting worse, as shown in Figure 2A. The surface roughness of liquid metal increases along with stirring time (Figure 2D and Supplementary Figure S4). After stirring for 60 min, its surface roughness reaches up to 0.409 μm.

Due to oxidation, the weight of liquid metal is also increased during magnetic stirring (Supplementary Figure S5). To analyze this variation, an X-ray photoelectron spectrometer is applied to characterize the surface composition of EGaIn before and after stirring. Figure 2C shows high-resolution spectra from 14 eV–21 eV, which includes the regions of Ga^0^, In4d, and Ga3d (Ga_2_O_3_). The peaks of Ga^0^ and In4d remain unchanged during the agitating process. Notably, stirred EGaIn shows a higher Ga3d ratio than the original EGaIn. Meanwhile, energy dispersive X-ray spectroscopy analysis shows that the ratio of oxygen elements increases from 0.8 %–5.4% (Figure 2E and Supplementary Figure S6). The results indicate that magnetic stirring boosts the degree of oxidation of the liquid metal. Hence, a liquid metal that is stirred for 60 min is named “O-EgaIn.” As shown in Figure 2F, the conductivity of untreated EGaIn is 4.5 × 10^6^ S/m, and O-EGaIn also maintains a high conductivity of 4.48 × 10^6^ S/m. Although the oxide layer of liquid metal is insulative, a small amount of oxide has no remarkable influence on conductivity. However, the addition of oxides might reduce the flexibility of liquid metal. As shown in Supplementary Figure S7, the original EGaIn surface stays flat after 200% stretching, while cracks gradually appear during the stretching test.

Adhesive forces between liquid metal and flat PDMS surfaces are investigated in Figure 2G. The adhesive force of the original EGaIn is about 0.3 mN, which is weaker than the adhesive force of stirred EGaIn (0.78 mN). As stirring time increases, EGaIn presents higher adhesion and smaller contact angle on a flat PDMS surface (Supplementary Figure S8). The change of wettability results from the mixed oxide particles. The wettability of EGaIn on a polymer surface is determined by intermolecular hydrogen bonding between methyl of the polymer and the oxide shell of EGaIn. The emergence of oxide particles promotes the formation of more intermolecular hydrogen bonds so that O-EGaIn shows higher adhesion on the polymer surface as stirring time increases. Meanwhile, abundant oxide particles reduce the fluidity of liquid metal and improve the stability of the O-EGaIn contact area. Hence, the obtained O-EGaIn has higher adhesion and better plasticity, which makes it a remarkable candidate for conductive ink. For instance, it can be directly brushed onto sandpaper, copper, and leaf, which are all difficult to print on by traditional processes (Figure 2H).

### 2.3 Controlling the wettability of O-EGaIn by an fs laser

**FIGURE 3:**
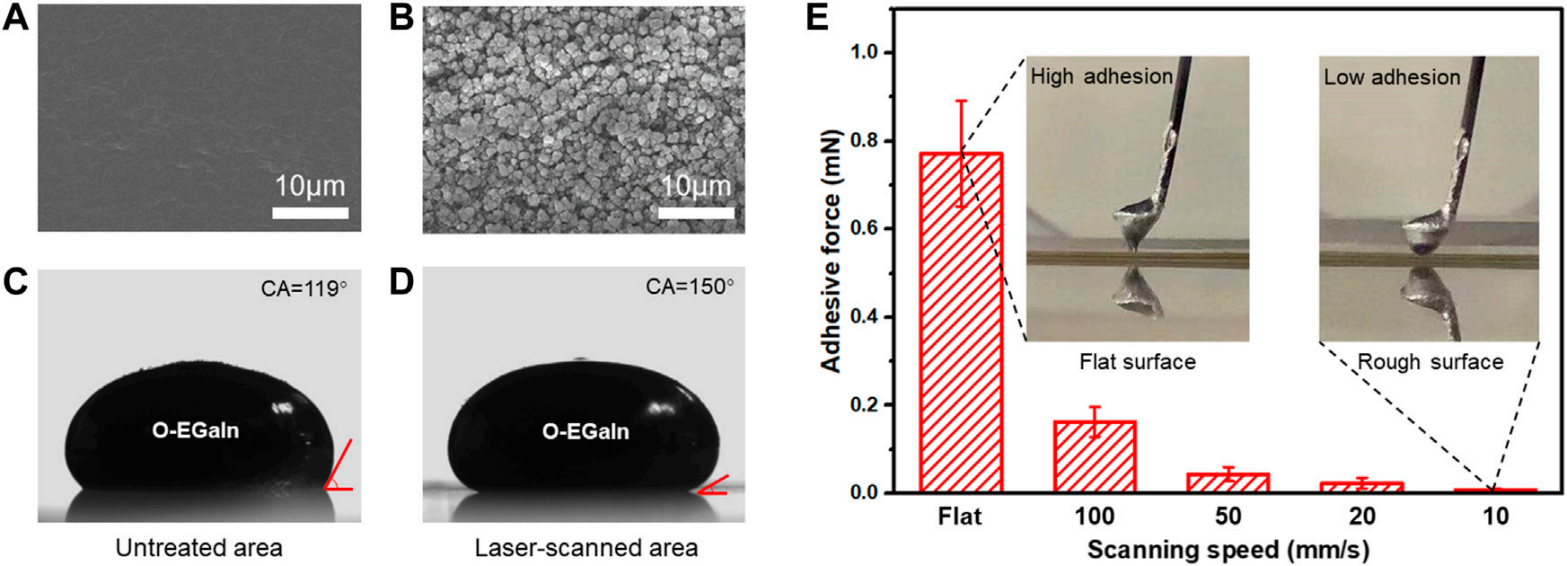
Wettability of O-EGaIn on different PDMS surfaces. **(A)** SEM image of the untreated PDMS surface. **(B)** SEM image of the fs laser-scanned PDMS surface. **(C)** O-EGaIn droplet on the untreated surface. **(D)** O-EGaIn droplet on the laser-scanned surface. **(E)** Adhesive force between O-EGaIn droplet and fs laser-scanned surface with different scanning speeds.

The surface wettability of liquid metal is determined by surface topography. Due to the higher adhesion of O-EGaIn, hierarchical nanostructures with high roughness are required in the liquid-metal-repellent area. Figure 3A shows a scanning electron microscope (SEM) image of a flat PDMS surface. After vibration to remove the yield stress of liquid metal, the contact angle of the O-EGaIn droplet (10 μL) is 119° (Figure 3C). Figure 3B shows that irregular coral-like sub-nanostructures are built on the PDMS surface after fs laser irradiation with a scanning speed of 10 mm/s. Eject nanoparticles coagulate and coat the surface, forming abundant smaller nanostructures with the size of hundred nanometers. The hierarchical structures greatly reduce the real contact area between O-EGaIn and the PDMS surface, and its contact angle is measured to be 150° after vibration (Figure 3D). The adhesive forces of O-EGaIn on fs laser-irradiated surfaces with different scanning speeds are depicted in Figure 3E. An O-EGaIn droplet is set on the cantilever in advance. Then, the O-EGaIn droplet slowly contacts the as-prepared surface and lifts. As shown in the insets of Figure 3E, O-EGaIn presents ultrahigh adhesion on a flat PDMS surface, while O-EGaIn has almost no deformation during leaving up the rough surface. As the scanning speed decreases, the real contact area is reduced by the surface roughness. The adhesive force changes from 0.16 mN (100 mm/s) and 0.04 mN (50 mm/s) to 0.02 mN (20 mm/s). When scanning speed is set at 10 mm/s, its adhesive force is only 0.002 mN, presenting ultralow adhesion to O-EGaIn. Therefore, scanning speed is chosen as 10 mm/s to realize a patternable liquid-metal-repellent surface.

### 2.4 Sacrificial layer assistant

**FIGURE 4:**
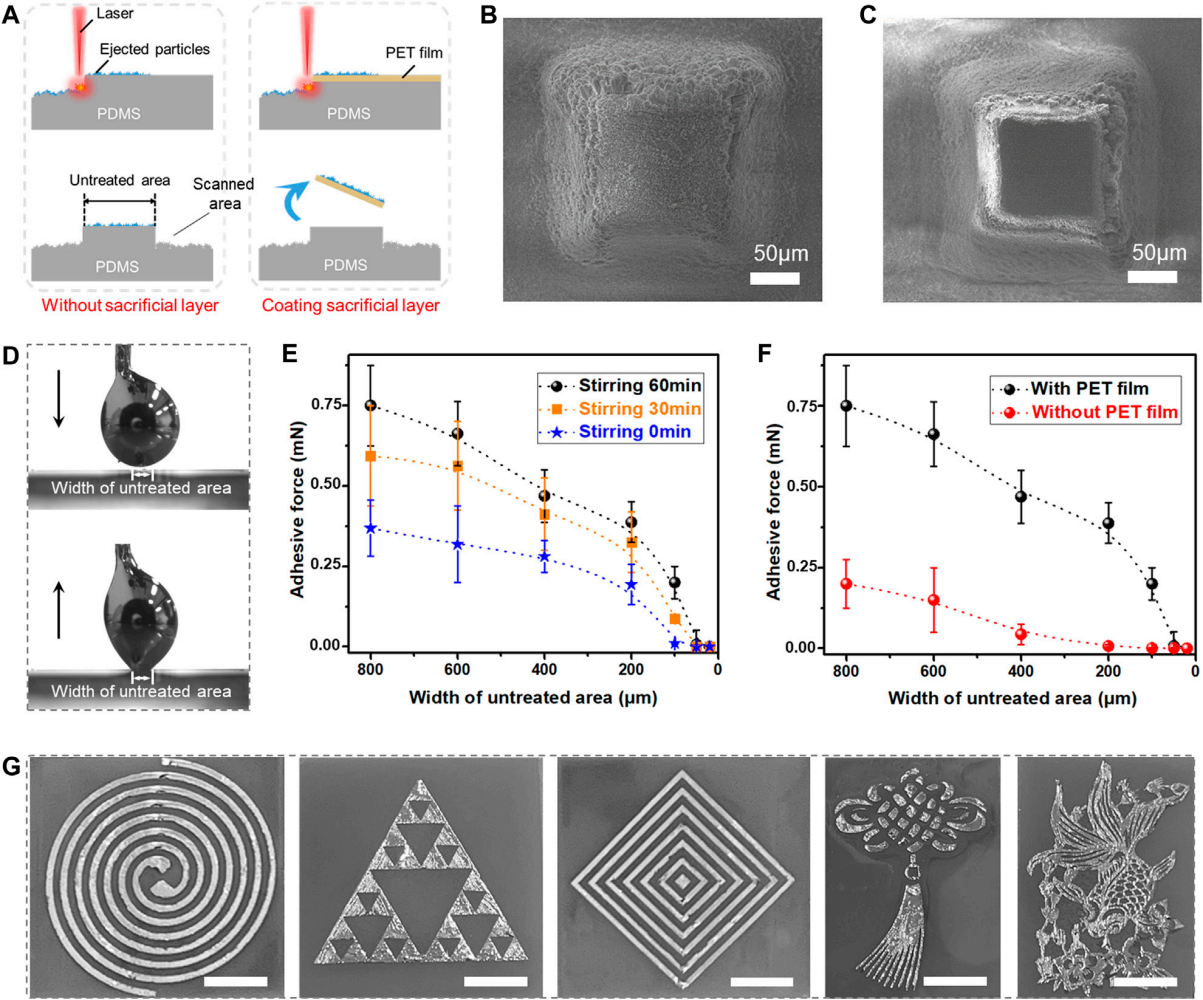
Influence of sacrificial layer on liquid metal printing. **(A)** Usage of the sacrificial layer to remove the ablation particles on an un-scanned area. **(B)** SEM image of the untreated square pattern on PDMS surface without sacrificial layer. **(C)** SEM image of the untreated square pattern using sacrificial layer method. **(D)** Process of a liquid metal droplet contacting with and then leaving from the untreated square pattern on PDMS surface with different widths. **(E,F)** Relationship between adhesive force and width of untreated area with **(E)** different stirring times and **(F)** different sacrificial layer treatments. **(G)** Optical images of LM patterns on PDMS surfaces with high resolution, such as spiral, triangular array, diamond array, Chinese knot, and goldfish. Scale bar: 5 mm.

Ideally, an fs laser is selectively ablated on the PDMS surface, so that O-EGaIn can be only wetted on an untreated smooth area, forming a liquid metal pattern. However, as illustrated in Figure 4A, laser-ejected nanoparticles not only coagulate on fs laser-ablated area but also adhere firmly to the edge of the untreated flat area, which is hard to clean. The ejected particles increase the surface roughness and prevent the real contact area between the liquid metal and the untreated surface. Thus, ejecting nanoparticles prevents further improvements in liquid metal printing resolution. To solve this problem, PET film is used as a sacrificial layer and coated on the PDMS surface during the fs laser scanning process. Since the thickness of the PET film is only 10 μm, a focused laser will remove the sacrificial layer and create micro/nanostructures on the laser-scanned area at the same time. For untreated areas, ejected particles are coated on PET film rather than the flat PDMS surface. After this process, the resultant surface is cleaned with alcohol for 10 min. The remaining PET film and coated nanoparticles are completely removed from the untreated area so that a flat PDMS surface is exposed. Figure 4B shows the untreated square pattern on the PDMS surface without the sacrificial layer assistant. Abundant nanoparticles are deposited on the untreated square area. Its surface roughness is 0.617 μm (Supplementary Figure S9). During sacrificial layer assistant, ejected nanoparticles are blocked by PET film. On this basis, the untreated PDMS surface stays smooth with a surface roughness of only 0.132 μm (Supplementary Figure S9).

To investigate the minimum width of our liquid metal printing method, smooth square patterns surrounding the laser-ablated area on PDMS surfaces are applied in this experiment (Figure 4C). As shown in Figure 4D, when an O-EGaIn droplet is put on the as-prepared PDMS surface and then left up, the droplet will be attracted only to a square area. Figure 4E indicates that the adhesive force decreases with the width of the square pattern reducing. The adhesive forces of O-EGaIn on flat square patterns are 0.75 mN (the width of 800 μm), 0.66 mN (the width of 600 μm), 0.47 mN (the width of 400 μm), 0.39 mN (the width of 200 μm), 0.20 mN (the width of 100 μm), 0.011 mN (the width of 50 μm), and 0.002 mN (the width of 20 μm), respectively. Thus, the narrower the width of the square area is, the more difficult it is for the liquid metal to leave a residue on the as-prepared surface. Meanwhile, the adhesive force increases with the stirring time of EGaIn. As the width is 100 μm, the original EGaIn presents an ultralow adhesion of 0.005 mN, which is even lower than the adhesion of O-EGaIn on the 50 μm wide square. The effect of the sacrificial layer is depicted in Figure 4F. In the absence of PET film, the adhesive forces of O-EGaIn on square patterns are reduced remarkably. The adhesive force is 0.20 mN (the width of 800 μm), 0.08 mN (the width of 600 μm), and 0.05 mN (the width of 400 μm), respectively. As the width decreases to 200 μm, its adhesive force is already smaller than 0.008 mN, showing ultralow adhesion. Notably, the printing effect of liquid metal on a narrow line is positively correlated with the measured adhesive force (Supplementary Figure S10). In the absence of a sacrificial layer, O-EGaIn is difficult to wet on a 200 μm wide line. The original EGaIn and sacrificial layer assistant can reach the resolution of 100 μm. By combining O-EGaIn and sacrificial layer assistant, a liquid metal pattern with a line width of 50 μm is achieved in the final. Due to the high accuracy, strong flexibility, and high efficiency of our fs laser scanning technology, any flat pattern can be fabricated by fs laser selective scanning. On this basis, various complex liquid metal patterns such as spiral patterns, triangle patterns, diamond-shaped patterns, Chinese knot patterns, and goldfish patterns can be successfully realized (Figure 4G).

### 2.5 The uniformity of as-prepared liquid metal electronics

**FIGURE 5:**
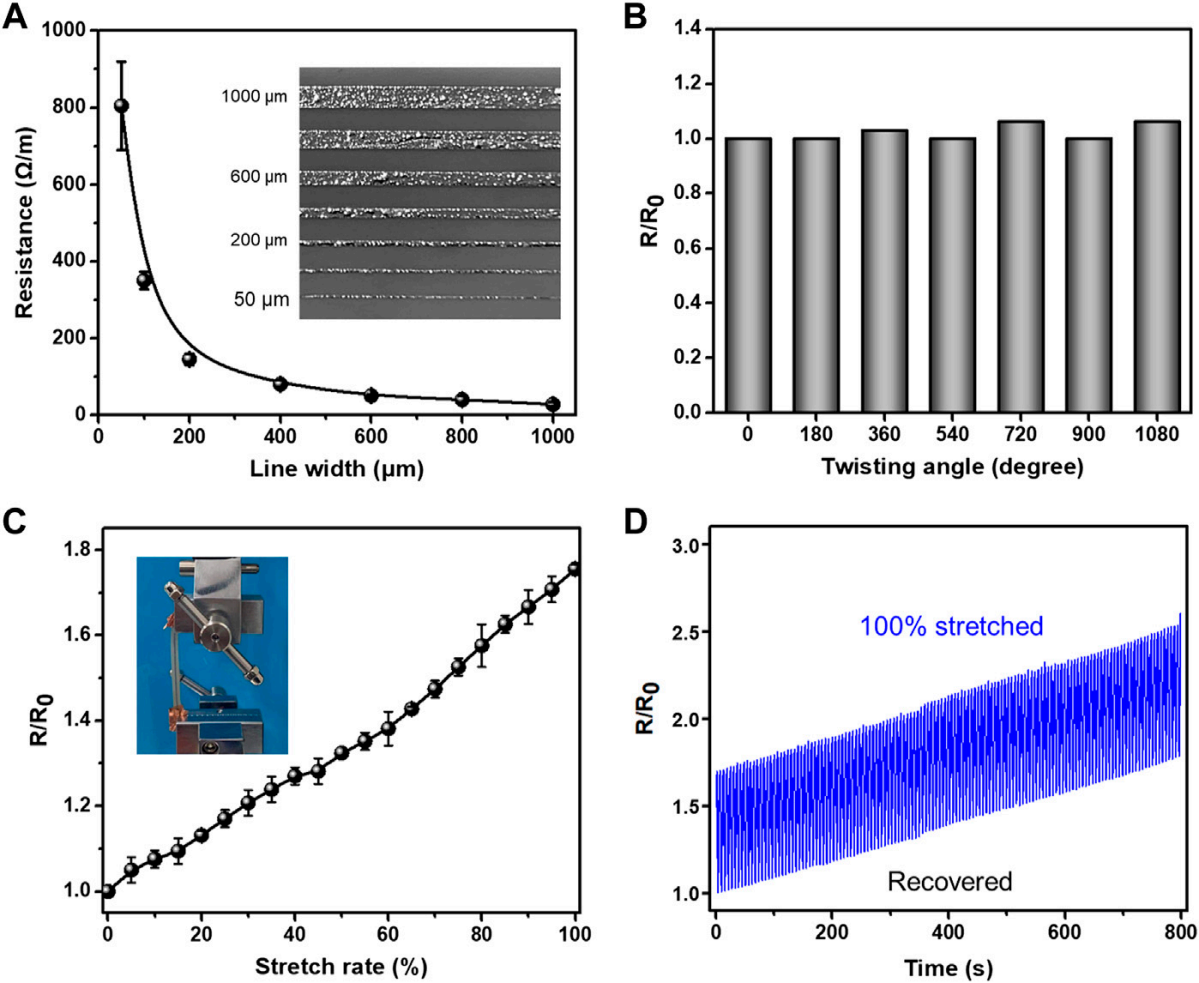
Conductivity of printed liquid metal lines. **(A)** Relationship between the resistance of liquid metal lines and their line widths. **(B)** Influence of the resistance on different twisting angles. **(C)** Variation of the resistance with stretch rate increasing. **(D)** Reversible switching of the resistance with repeated stretching and recovering.

To verify the uniformity and connectivity of the liquid metal pattern, liquid metal lines with different widths are printed on the PDMS surface for flexible electronics. As shown in Figure 5A, when the widths of O-EGaIn lines decrease from 1,000, 800, 600, 400, 200, 100, and 50 μm, their resistances are measured as 27.5, 40, 50, 80, 145, 350, and 805 Ω/m, respectively. The relationship between the line width and its resistance is approximate in inverse proportion, presenting the uniformity of printed liquid metal lines. Then, the liquid metal line is twisted from 0° to 1,080° while its resistance has little change during the twisting test (Figure 5B). The change in the resistance with different stretch rates is described in Figure 5C. R/R_0_ is approximately in direct proportion to the stretch rate. When the s stretch rate is controlled at 100%, R/R_0_ reaches up to 1.76. In addition, the liquid metal line on the PDMS substrate is stretched and recovered for 300 cycles (Figure 5D). Although its resistance slightly increases with stretching cycles, the change is no more than 10% after a 100% stretch for 30 times. Hence, the as-prepared liquid metal lines present a good, stable conductivity during these tests.

**FIGURE 6:**
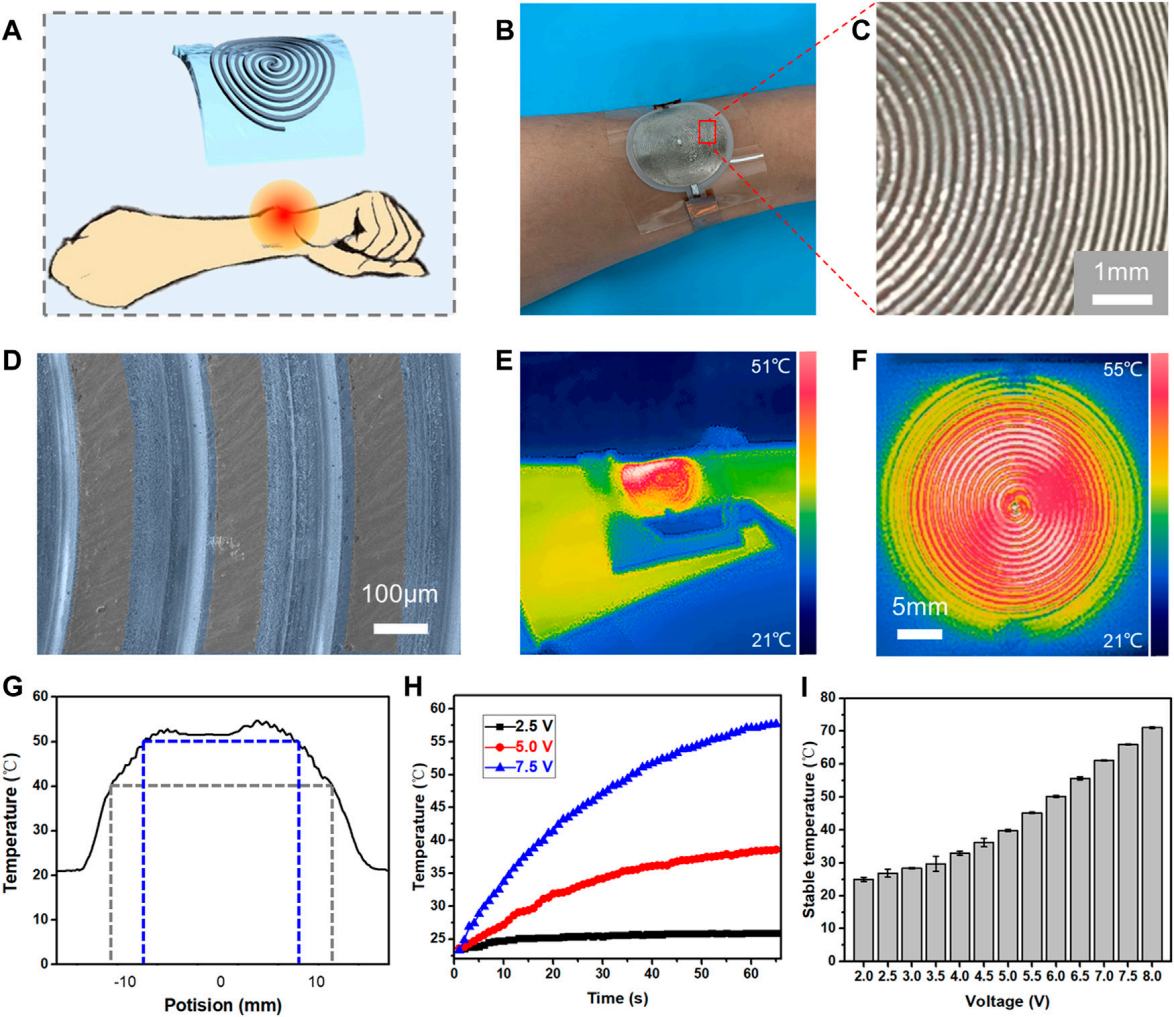
Uniformity of laser-induced flexible microheater *via* combining O-EGaIn and the sacrificial layer. **(A)** Schematic of the electronic skin to control the local temperature of the body. **(B)** Photograph of as-prepared flexible microheater. **(C)** Uniformity of the liquid metal line. **(D)** SEM image of the liquid metal spiral line. **(E,F)** Infrared temperature distribution image of the flexible microheater. **(G)** Temperature field uniformity test of microheater. **(H)** Change in temperature of the microheater as a function of time. **(I)** Relationship between the input voltage and the stable temperature of the microheater.

Meanwhile, liquid metal spiral lines are prepared on the PDMS surface and applied as electronic skin (Figures 6A, B). The diameters of semi-circulars are changed from 1 mm to 30 mm, and both ends of the spiral lines connect with copper tape electrodes. As shown in Figure 6C, the obtained liquid metal lines are regular and uniform. Both the width of spiral lines and the distance of adjacent liquid metal lines are controlled at 100 μm. An SEM image captures that O-EGaIn selectively wets on the untreated area of the PDMS surface, forming a liquid metal spiral line. The error in line width is less than 10 μm (Figure 6D and Supplementary Figure S11). As the electrode is powered on, the electronic skin will be heated continuously even under bending conditions, and its resistance is stable at 52 ± 6 Ω (Figure 6E). Figure 6F shows a thermal infrared image of O-EgaIn-based electronic skin, which is operating at the voltage of 5 V for 60 s. The red color is entirely distributed on the liquid metal printed area, meaning that there is no short failure or open failure in any part of the circuit. The distribution of the temperature field is analyzed in Figure 6G. The temperature of the whole liquid metal printed area is higher than 40°C, and the highest temperature is found to be 54.8°C. In the range from −8 mm to 8 mm, the temperature stays higher than 50°C, and its difference is less than 5°C. The uniformity of the temperature distribution demonstrates the accuracy of our fs laser-induced liquid metal printing technology.

Temperature control of the electronic skin is also being explored. According to Joule’s law, the heating rate can be controlled by adjusting the input voltage. As shown in Figure 6H, the heating rate increases with input voltage. Then, the rate of heating is gradually slowed down, and the temperature stays stable in the end. Figure 6I shows that input voltage is positively associated with stable temperature. The stable temperature of 28.4°C–50.2°C can be realized by setting the input voltage from 3 V to 6 V. It is mentioned that the flexible liquid metal spiral line can meet the requirements of wearable electronics for local heating by pasting on body skin and clothes. Body temperature (36.2°C) is realized by controlling the voltage at 4.5 V, which is much lower than the safe voltage for humans. Therefore, the fs laser-induced liquid metal spiral line has high uniformity and is expected to integrate into electronic skin to control the body temperature.

## 3 Conclusion

We propose a strategy to realize complex liquid metal patterns with high resolution *via* combining O-EGaIn, fs laser, and sacrificial layer assistant. To adhere to a flexible substrate firmly, liquid metal is partly oxidized by a simple stirring process. Its adhesive force on flat PMDS can be reached up to 0.3–0.78 mN. To control the liquid metal printed area, a fs laser is utilized for selective scanning on the PDMS surface. Hierarchical nanostructures are built on laser-scanned areas, presenting super-lyophobicity to O-EGaIn, while untreated areas remain ultrahigh adhesion to liquid metal. Taking advantage of the sacrificial layer, the influence of laser-ejected nanoparticles is also eliminated. Therefore, O-EGaIn can be selectively wetted on an as-prepared surface, forming liquid metal patterns. The resolution can be controlled even less than 50 μm after optimization. The resultant liquid metal pattern is applied to wearable electronics and presents uniformity, flexibility, and stability. Compared with other liquid metal printing methods, an accurate O-EgaIn pattern is realized with no need for dopants and printing masks. We believe the high-resolution liquid metal printing method will make a contribution to liquid metal-based electronics, such as electronic skins, mobile communication, extreme manufacturing, and wearable electronics.

## 4 Experimental section

### 4.1 Materials

PDMS substrates with a thickness of 3 mm were bought from Hangzhou Bald Advanced Materials Co., Ltd. EgaIn (70% Ga & 30% In) was purchased from Wochang Metal Co., Ltd. To obtain O-EgaIn, pure EgaIn was directly stirred on a magnetic stirrer at room temperature without any chemical treatment. PET thin films (Darit Tape Co., Ltd.) with a thickness of 10 μm were chosen as the sacrificial layer.

### 4.2 Femtosecond laser scanning

Fs laser was utilized to irradiate the PDMS surface. The laser beam (50 fs, 800 nm, 1,000 Hz) was generated from a Ti:sapphire laser system (Coherent, Librausp 1K-he200) and focused by a telecentric lens (NV13114855, Scanlab, Germany). The laser-irradiated pattern was controlled and programmed by a high-speed scanning galvanometer (SL2-100, Scanlab, Germany). The spot diameter of the focused fs laser beam was about 25 μm, and its focal depth was 60 μm (Supplementary Figure S1). Laser-ablated width was determined by laser power and scanning speed (Supplementary Figure S12). In this work, a line-by-line fs laser scanning process is used for laser ablation (Yong et al., 2020). Laser power was set at 300 mW. The adjacent scanning line was controlled as 10 μm, and the scanning speed was 10 mm/s.

### 4.3 Characterization

3D confocal microscopy images of liquid metal and their surface roughness were obtained by a laser confocal microscope (OLS4000, Olympus, Japan). The variation of the oxygen content of EGaIn was characterized by an X-ray photoelectron spectroscopy (Escalab Xi +, Thermo Fisher, America) and an energy dispersive X-ray spectroscopy (Model 550i, IXRF Systems, America). The four probe method was utilized for measuring the conductivity of liquid metal. Adhesive forces between liquid metal and PDMS surfaces were investigated by a surface tension measurement system (Dact 11, Dataphysics, Germany). The surface morphologies of PDMS substrates were captured by a scanning electron microscope (Flex 1,000, Hitachi, Japan). A contact angle system (JC 2,000D, Powereach, China) was utilized for measuring the contact angle of EGaIn and O-EGaIn droplets. The temperature variation of the microheater was observed by a thermal infrared camera (M300, InfiRay, China).

## Data Availability

The original contributions presented in the study are included in the article/Supplementary Material; further inquiries can be directed to the corresponding author.
