# Supplementary material for: Controlling the oxidation and wettability of liquid metal via femtosecond laser for high-resolution flexible electronics
Source: Front Chem. 2022 Sep 1;10:965891. doi: 10.3389/fchem.2022.965891 (PMC9475219; doi:10.3389/fchem.2022.965891)
Supplement: Supplementary file 1 [file DataSheet1.docx]

Controlling the Oxidation and Wettability of Liquid Metal via Femtosecond Laser for High-Resolution Flexible Electronics

**Jingzhou Zhang^1^, Chengjun Zhang^2^，Haoyu Li^1^, Yang Cheng^2^, Qing Yang^2^, Xun Hou^1^, and Feng Chen^1,*^**

*^1^ State Key Laboratory for Manufacturing System Engineering and Shaanxi Key Laboratory of Photonics Technology for Information, School of Electronic Science and Engineering, Xi’an Jiaotong University, Xi’an, 710049, PR China*

*^2^ School of Mechanical Engineering, Xi’an Jiaotong University, Xi’an, 710049, PR China*

E-mail: [chenfeng@mail.xjtu.edu.cn](mailto:chenfeng@mail.xjtu.edu.cn)


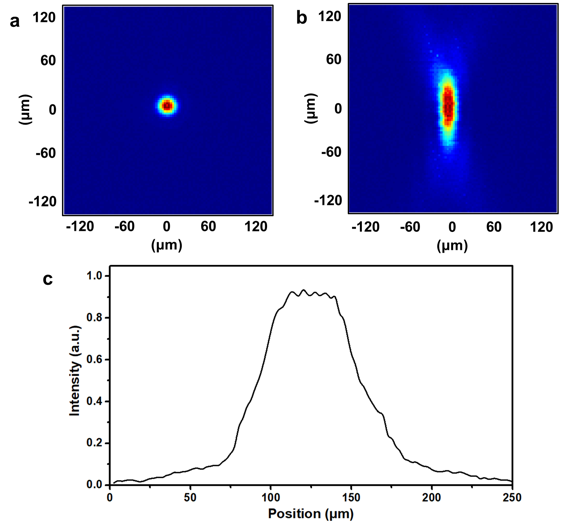


**Figure S1.** (a) Radial intensity distribution of the laser beam. (b) Longitude intensity distribution of the laser beam. (c) The energy distribution of the center spot beam along the propagation direction.


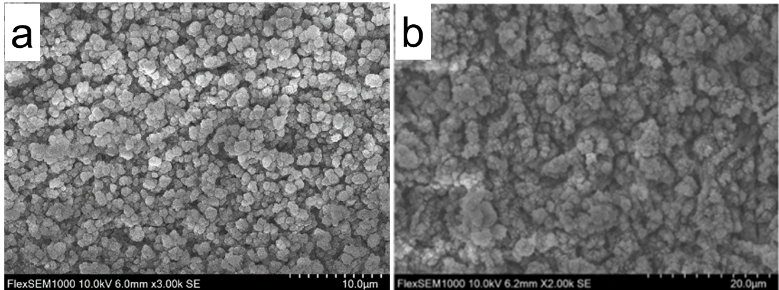


**Figure S2.** (a) SEM image of laser-ablated PDMS surface with PET film. (b) SEM image of laser-ablated PDMS surface without PET film.


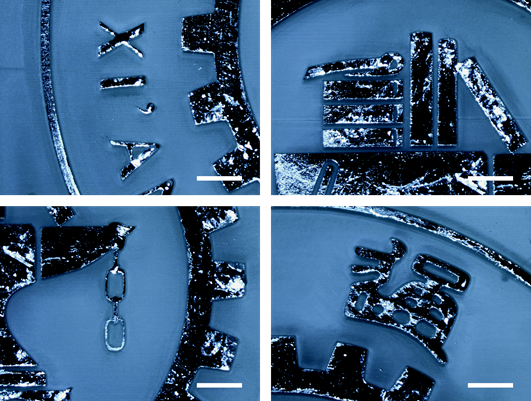


**Figure S3.** The optical microscope images of the liquid metal logo with higher magnification. Scale bar is 2 mm.


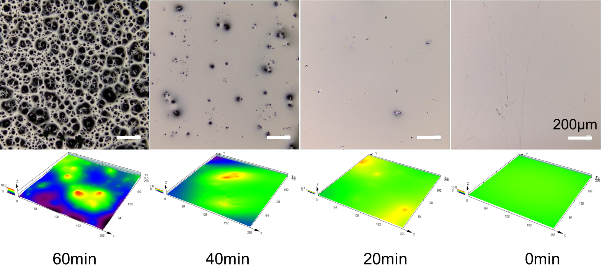


**Figure S4.** Optical and 3D confocal microscopy images of EGaIn surfaces with different stirring time.


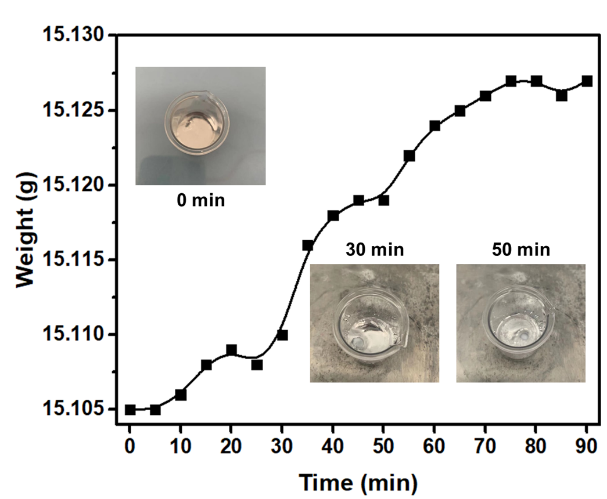


**Figure S5.** The weight of EGaIn with different stirring time.


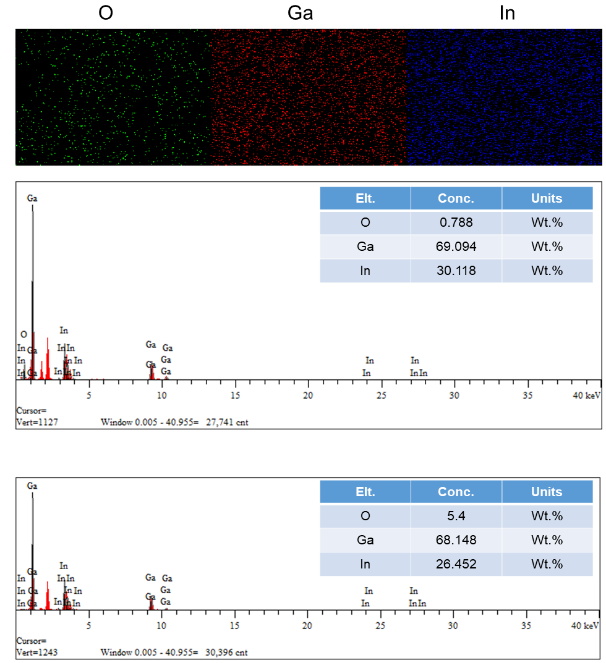


**Figure S6.** EDXS result of the untreated EGaIn and stirred EGaIn.


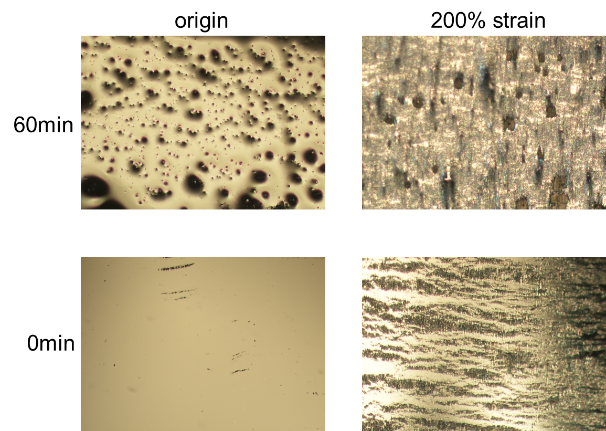


**Figure S7.** Optical images of EGaIn surfaces before and after straining.


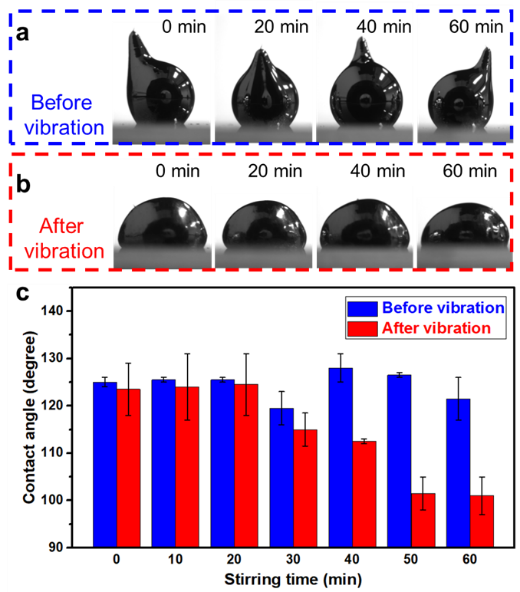


**Figure S8.** The wettability of magnetic stirred EGaIn before and after vibration with different stirring time.


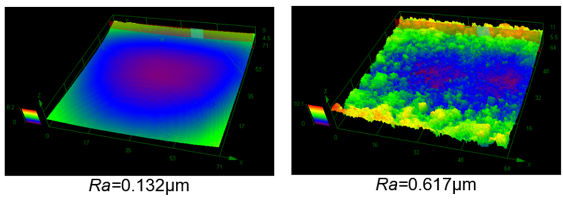


**Figure S9.** 3D confocal microscopy images and surface roughness of edge of the untreated PDMS surface using sacrificial layer (left) and without sacrificial layer (right).


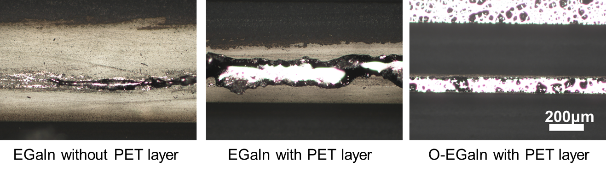


**Figure S10.** Printing effect of liquid metal on different PDMS surfaces.


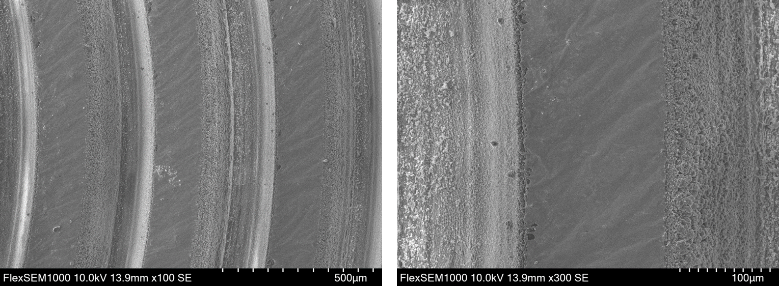


**Figure S11.** Original SEM images of liquid metal spiral lines.


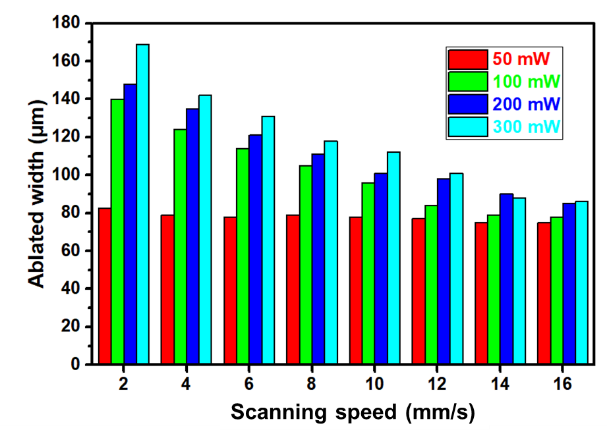


**Figure S12.** Ablated width of the laser scanned line with different scanning speed and laser power.
